# Health care seeking behaviour and utilisation in a multiple health insurance system: does insurance affiliation matter?

**DOI:** 10.1186/1475-9276-13-25

**Published:** 2014-03-19

**Authors:** Eunice Nahyuha Chomi, Phares GM Mujinja, Ulrika Enemark, Kristian Hansen, Angwara Dennis Kiwara

**Affiliations:** 1 Muhimbili University of Health and Allied Sciences, Dar-es-Salaam, Tanzania; 2 Aarhus University, Aarhus, Denmark; 3 London School of Hygiene and Tropical Medicine, London, UK

**Keywords:** Health insurance, Health care utilisation, Health seeking behaviour, Equity

## Abstract

**Background:**

Many countries striving to achieve universal health insurance coverage have done so by means of multiple health insurance funds covering different population groups. However, existence of multiple health insurance funds may also cause variation in access to health care, due to the differential revenue raising capacities and benefit packages offered by the various funds resulting in inequity and inefficiency within the health system. This paper examines how the existence of multiple health insurance funds affects health care seeking behaviour and utilisation among members of the Community Health Fund, the National Health Insurance Fund and non-members in two districts in Tanzania.

**Methods:**

Using household survey data collected in 2011 with a sample of 3290 individuals, the study uses a multinomial logit model to examine the influence of predisposing, enabling and need characteristics on the probability of seeking care and choice of provider.

**Results:**

Generally, health insurance is found to increase the probability of seeking care and reduce delays. However, the probability, timing of seeking care and choice of provider varies across the CHF and NHIF members.

**Conclusions:**

Reducing fragmentation is necessary to provide opportunities for redistribution and to promote equity in utilisation of health services. Improvement in the delivery of services is crucial for achievement of improved access and financial protection and for increased enrolment into the CHF, which is essential for broadening redistribution and cross-subsidisation to promote equity.

## Background

Health insurance has emerged as a key instrument in current health financing reforms of middle and low income countries aimed at achieving universal coverage. The health insurance systems of these countries are often characterised by multiple health insurance funds covering different population groups. When mechanisms to promote cross-subsidies across funds exist within the health insurance system, the risk pools are referred to as integrated. Without such mechanisms the risk pools are referred to as fragmented [1]. Arguably, using multiple health insurance funds is the most practical means with which to achieve universal coverage given the constraints of enforcing universal mandatory coverage [2]. However, when the risk pools are fragmented, this also causes variation in the potential access, health care seeking behaviour and utilisation of health services. This is likely because apart from reducing the financial barriers associated with the cost of health services, health insurance also influences health care seeking behaviour (whether, when, from where care is sought for an illness) by preventing delays, self-treatment and use of alternative forms of care [3]. In addition, structural features of the health insurance system such as contribution levels, eligibility and benefit entitlements determine who is covered as well as the quality, type and quantity of services covered. Hence the way health insurance system is organised is likely to influence health care seeking behaviour and utilisation of health services. Also, the differential revenue raising capacities and benefit packages offered by the various insurance funds in a fragmented system are likely to result in varying degrees of access, health care seeking behaviour and utilisation of health services [1,4]. Furthermore, fragmentation results in inefficiently high administrative costs, which may have an impact on the ability of the health system to achieve its policy objectives of financial protection [5,6].

Existence of fragmentation also results in a tiered health system, which is inequitable [2]. The experience of many Latin American countries exemplifies this, where social health insurance for formal workers co-existed with national health services delivered directly through ministries of health to provide the poor and informal workers with health service coverage. This led to a tiered system whereby formal sector workers enjoyed access to a wide range of high quality services while the rest had access to a less generous benefit package while incurring higher costs due to co-payments or excluded services [7-10]. Thus, health insurance systems with fragmented risk pools lack the necessary conditions for cross-subsidisation to promote financial protection and equitable utilisation of health services [11].

Tanzania has two predominant health insurance funds, the Community Health Fund (CHF) and the National Health Insurance Fund (NHIF). The NHIF is mandatory for public sector employees covering 7.2% of the population while the CHF is voluntary and district based for the rural population with coverage of about 6.6% [12]. Other insurance funds include an urban equivalent of CHF for the informal population,  *Tiba Kwa Kadi*’ (TIKA) and the Social Health Insurance Benefit (SHIB) for members of the National Social Security Fund (NSSF). There are also various private health insurance funds mostly covering those in the formal sector through their employers and micro-insurance schemes which cover mostly informal sector workers [13,14].

The health insurance system is fragmented, with no transfers between the risk pools despite the differential health care needs and revenue bases. NHIF members are entitled to a relatively comprehensive package of health services which include specialised services that can be accessed from government and accredited private primary, secondary and tertiary care providers. In contrast CHF members are entitled to a package of health care services which they can access from primary care providers [13]. This implies that members of CHF and NHIF have varying degrees of access to health care hence it is likely that this may influence their health care seeking behaviour and utilisation. While some degree of cross-subsidisation between the healthy and the sick occurs within the CHF and NHIF, the lack of cross-subsidies across the funds limits the extent to which resources can be redistributed to promote equitable utilisation.

Against this background, this paper intends to examine the effects of fragmented risk pooling on health care seeking behaviour and utilisation of CHF and NHIF members and non-members in two districts in Tanzania. Specifically we aim to examine the differences in health care seeking behaviour and utilisation between CHF, NHIF and non-members. Bivariate and multivariate analyses are used to study the relationship between membership status and the decision and timing to seek care, and choice of provider given a set of predisposing, enabling and need characteristics. The remainder of this section provides a discussion of a framework for health care utilisation. Section two describes the methods and the data used in this study and section three presents the results. Section four discusses the results and the final section provides some conclusions of the study in terms of policy and further analysis.

### Health care seeking behaviour and utilisation

One of the most frequently used frameworks for the analysis of health care utilisation is Andersen’s behavioural model of health care use [15-21]. This framework assumes that utilisation of health care is influenced by the predisposition, the ability and the need to use health services [22,23].

Predisposing factors relate to the propensity to utilise health services and include individual characteristics that are not directly related to health care utilisation but rather influence the likelihood of utilisation. These characteristics can be categorised as: demographic, social structure and health beliefs [24]. Demographic characteristics include age and sex, which represent biological factors that affect the likelihood that an individual will need health services. Social structure represents the factors that determine the status of an individual in the society as well as the physical and social environment. The most common measures of social structure are education level, occupation and ethnicity. Health beliefs are the attitudes, values and knowledge that an individual may have about health and health services that may influence utilisation of health services [23].

Enabling characteristics describe the means that individuals have at their disposal with which to utilise health services. This is based on the argument that even though an individual may be predisposed to utilise health services, certain factors must be in place to enable actual use. These include income, health insurance status and availability of health services. Usually residence (urban/rural) and distance are used as proxy measures for availability of health services. Need characteristics are the direct determinants of health care use which include self reported and evaluated morbidity [22].

Health insurance is the primary variable of interest in this study, due to the key role in improving access to health care by reducing financial barriers to utilisation of health services. It is therefore expected that health insurance should positively influence the probability of utilisation. The fragmentation of the health insurance system and the differential benefit packages between the CHF and NHIF implies that members of these two funds will have differential access to health care, which will inherently influence their choice of provider. From this perspective, we expect that choice of provider will be influenced by insurance affiliation. Predisposing and need variables are used as control variables.

## Methodology

### Study setting, design and data collection

Data for this study was obtained from Kongwa and Mpwapwa districts in Tanzania over a period of eight weeks between July and September 2011. The two districts were selected due to their different levels of CHF enrollment, and for convenience in terms of logistics and costs. Kongwa has a total of 63,612 households of which 5,800 (9%) are registered with CHF [25]. Mpwapwa has a total of 78,812 households of which 15,540 (18%) are registered with CHF [25]. The prime economic activity in both districts is agriculture and livestock keeping.

### Sampling method and sample size calculation

For the purposes of this study a household is defined as a person or group of people related or unrelated who live together and share a common pot of food and who share the same membership card (for CHF) or are dependents of the same principal member^a^ (NHIF households). This was adapted from the 2010 Tanzania Demographic and Health Survey (TDHS) definition of a household [26]. The study population comprised of all individuals living in the households in the two districts which met this definition. In each district a multi-stage sampling approach was used to select first wards, then villages followed by hamlets^b^ and eventually households. Due to difficulties in identification of households by membership status from the village household register^c^, equal numbers of households were selected from listings of each membership category as follows: CHF households were randomly selected from the CHF register book kept in the health facilities in each ward. This was because health facilities are registration points for CHF registration. The health facilities were selected based on whether the facility catchment area falls within the selected hamlets. The selection was made from members registered from September 2010 to September 2011, to ensure only current CHF members were included.

For NHIF households, a list of all Government institutions in the selected wards or villages was obtained from the District Council, from which all available (at the time of the study) NHIF principal members were selected. This approach was used since there are few NHIF members. Non-member households were randomly selected from the village household register in each of the selected villages. All CHF and NHIF households were omitted from the village register using the list obtained from the facility and District Council respectively before selection of non-member households. In each household all members were interviewed.

Data used for analysis was collected as part of a larger study, hence estimated sample size calculations were based on all the key study variables and the maximum sample size estimate was used, since this would be sufficient for the analysis of all key variables [27]. Hence, while we obtained estimates based on the key variables for this analysis, they were not sufficient for the analysis of other key variables. The sample size calculation was based on the assumptions that there would be 80% power to detect a 25% difference between CHF and NHIF households in the number of facility visits per year. We used the proportion from a similar study, which reported about 50% of insured households having at least one facility visit per year [28] and assumed the proportion of NHIF households to be higher. This resulted in a sample of 729 households (243 per group). Estimating a non-response of 5%, final sample size was adjusted to 766 households. Using the average household size of 5 persons reported by the 2010 TDHS, this represents a sample size of approximately 3830 individuals.

A pre-tested structured questionnaire was administered to the household head or spouse. Data was collected on demographic characteristics, employment, education level, family size, membership status, household ownership of assets and consumer durables, presence of chronic and acute illnesses, general health status, health care seeking behaviour and utilisation of health services. Three return visits were made to households where members were not available for interview during the first visit, resulting in a response rate of 85%, with a sample size of 3290 individuals from 695 households.

### Study variables

For the bivariate analysis, the decision and timing of seeking care was compared across the CHF, NHIF and non-members. The decision to seek care relates to whether or not care was sought for an illness experience during the four weeks recall period. Timing relates to the time elapsed between the onset of symptoms of illness and seeking care (same day, less than 1 week, more than 1 week).

For the multivariate analysis, choice of provider, defined as the place of first contact following an illness during the four weeks recall period (public hospital, private health facility, public health centre/dispensary or traditional healer/self medication) was the dependent variable. The alternative of traditional healer or self medication refers to those who sought treatment outside the home from a traditional healer, drugstore or pharmacy. This differs from individuals who did not seek care but instead used home remedies or those who delayed seeking care and opted to start with home remedies first. Traditional healer and self medication was later merged since both choices represent alternative sources of care. In addition the choice of traditional healer accounted for only 1% of those seeking care.

Drawing on Anderson’s 1995 model [23], independent variables used in this study include predisposing, enabling and need characteristics of individuals. Predisposing characteristics included age (0–5, 6–14, 15–49, 50–59, 60+ years), sex (male, female) and education level of household head (no education, primary education, secondary education, above secondary education). Perceived adequacy of staff and perceived availability of drugs (yes, no) were included as proxies for attitudes towards health services as expressed from general questions on health status and utilisation.

Enabling characteristics included were household characteristics that were assigned to an individual according to the household to which he/she belonged. These were membership status (CHF, NHIF, non-members), residence (urban, rural), distance to facility (less than or more than 5 km) and wealth status (lowest to highest wealth quintile). Owing to the complexities of determining actual income, Principal Components Analysis (PCA) was used to develop an asset index that grouped households into quintiles based on ownership of assets and durable goods [29,30]. The asset index of the household was used to represent socio-economic status. For the need characteristics we used self reported illness, defined as the experience of illness or injury lasting for a month or less (acute) or experience of an illness lasting for three months or more (chronic).

### Analysis

Bivariate and multivariate analysis was used to study the relationship of predisposing, enabling and need characteristics and health care seeking behaviour and utilisation. Chi square tests were used to study the relationship between membership status and health care seeking behaviour along two dimensions: the decision to seek care for an illness and timing. Multinomial logistic (MNL) regression was used to estimate the choice of provider given a set of predisposing, enabling and need characteristics. This model was selected based on the nature of the dependent variable and the ability of the model to estimate all choices in a single equation [31,32]. Since the aim was to find out whether insured individuals will choose a provider where they can use their insurance card, the health centre/dispensary category was used as a reference (both CHF and NHIF members can use their cards at this level).

The MNL model assumes that the odds of choosing between two alternative choices do not depend on which other choices are available (the Independence of Irrelevant Alternatives (IIA) assumption) [33]. We employed Hausman-McFadden (HM) and Small-Hsiao (SH) tests to validate this assumption. Both tests returned non-significant results (HM-ρ=0.112; SH-ρ=0.112), indicating that the model is appropriate. The use of alternative models that relax the IIA assumption such as the multinomial probit, nested logit and mixed logit models is limited by their computational difficulties and for the multinomial probit, the need for a particular data structure [32].

It is also possible that the same factors that influence health care utilisation could also influence the purchase of health insurance, implying that the health insurance variable is endogenous [33-35]. The mandatory nature of NHIF and household basis of membership for the CHF reduce the effect of selection bias in our study. However, possible endogeneity of the health insurance variable was tested using the Durbin-Wu-Hausman test, which uses an instrumental variable to test whether the predictors are correlated with the error term. A non-significant test result indicates that none of the predictor variables are endogenous [36]. We used relationship to the head of household as the instrumental variable and the test was not significant (ρ=0.315) implying that the health insurance variable was exogenous. Choice of the instrumental variable was based on its influence on health insurance membership but not on health care utilisation, which is a criterion for instrumental variable selection. Being related to the household head makes one eligible for insurance membership, but does not influence whether or where care will be sought for an illness.

Since individuals in the sample were obtained from households, the observations of each individual are not independent of each other, resulting in an under-estimation of standard errors and making significance tests used in the analysis invalid. The effect of clustering has been accounted for in the analysis, using clustered robust standard errors which increase the variability between individuals within cluster [37-39].

### Ethical considerations

Ethical approval was sought from the Research and Ethics Committee of Muhimbili University of Health and Allied Sciences. Following ethical approval, permission to conduct data collection was obtained from the Regional Administrative Secretary (RAS) of Dodoma and District Administrative Secretary (DAS) of Kongwa and Mpwapwa. Respondents were informed of the research objectives and were asked to participate in the study. Those who agreed were asked to sign a consent form.

## Results

### Descriptive characteristics of the sample

Table 1 presents the descriptive characteristics of the study sample. About 28% of the respondents were from NHIF households, 38% from CHF households and 34% from non-member households. More than 50% of NHIF members were in the 15–49 year age group, with less than 10% aged 0–5 years and less than 3% aged 60 years and above. In contrast 43% each of CHF and non-members were aged 15–49 years, 15% aged 0–5 years and 4% aged 60 and above (ρ<0.05). A higher proportion of CHF households (44%) had more than five members, compared to NHIF (28%) and non-member households (31%, ρ<0.05).

**Table 1:** Individual characteristics by membership status, Kongwa and Mpwapwa 2011 (%)

**Variable**	**NHIF**	**CHF**	**Non-members**
	**N = 931**	**N = 1242**	**1117**
Age***			
0-5	9.1	14.5	14.8
6-14	25.2	33.4	31.3
15-49	55.2	42.9	42.9
50-59	8.7	4.9	6.5
60+	1.8	4.3	4.4
**Sex**			
Male	47.1	48.6	46.9
Female	52.9	51.5	53.1
Education (head)***			
No education	1.4	20.7	30.7
Up to Primary	8.1	71.9	63.9
Up to–Secondary	27.9	6.5	5.0
Above secondary	62.6	0.7	0.4
Household size***			
Mean = 4.7	4.0	5.2	4.7
1-5 members	72.3	56.2	69.3
>5 members	27.7	43.8	30.7
Wealth Status***			
Lowest	2.5	29.4	33.2
Second	3.7	30.8	27.2
Third	14.7	22.7	22.7
Fourth	40.2	12.7	10.3
Highest	38.9	4.4	6.5
**Distance to facility**			
1-5 km	91.9	95.4	95.4
>5 km	4.6	8.1	4.7

***ρ<0.01; based on Chi square test.

NHIF head of households were more educated, with 63% having attained secondary or above secondary education, while the majority of CHF (72%) and non-members (64%) attained primary education (ρ<0.05). The majority of NHIF households are relatively wealthy (39% highest, 40% fourth wealth quintile) compared to CHF (29% lowest, 31% second wealth quintile) and non-member households (33% lowest, 27% second wealth quintile, ρ<0.05).

### Health care seeking behaviour and utilisation

Table 2 illustrates health care seeking behaviour by membership status. During the recall period of four weeks prior to the survey, 30 percent reported having had at least one spell of illness. Of those individuals, 75 percent reported having sought care for their illness.

**Table 2:** Health seeking behaviour by membership status, conditioned on reporting illness (%)

**Variable**	**NHIF**	**CHF**	**Non-members**
	**N = 931**	**N = 1242**	**N = 1117**
Reported illness (yes)	31.3	30.5	28.6
Sought care for illness?***	**N = 291**	**N = 379**	**N = 320**
Yes	81.1	77.3	65.6
No	18.9	22.7	34.4
Timing of care**	**N = 236**	**N = 293**	**N = 201**
Same day	66.1	57.3	52.7
<1 week	31.4	37.8	40.8
>1 week	2.5	4.8	6.5

**ρ<0.05; ***ρ<0.01; based on Chi square test.

#### ***Delays in seeking care***

The majority of CHF (57%) and NHIF (66%) members were more likely to seek care on the same day they fell ill, while more than 40% of non-members were more likely to experience delays in seeking care (ρ<0.05 ).

The reasons for delaying to seek care and/or not seeking care are presented in Table 3. Among those who delayed seeking care, the most common reason reported was “wait and see if illness progresses further” for NHIF (40%) and CHF (44%) members. Among non-members lack of money to pay for treatment was the main reason, reported by 34%. Reasons for not seeking care differed significantly by membership status. The main reasons were availability of own medicine/home remedies, reported by 43% of NHIF and 38% of CHF members and lack of money to pay for treatment, reported by 51% of non-members (ρ<0.05).

**Table 3:** Reasons for delays and/or not seeking care by membership status, conditioned on reporting illness (%)

**Variable**	**NHIF**	**CHF**	**Non-members**
Reasons for delays***	**N = 77**	**N = 120**	**N = 90**
No money	9.1	9.2	40.0
Distance	6.5	1.7	1.1
Self treatment (home)	18.2	16.7	12.2
“Wait and see”	49.4	49.2	33.3
Facility closed	10.4	11.7	10.0
Other	11.7	11.7	3.3
Reasons for not seeking care***	**N = 51**	**N = 68**	**N = 97**
Poor quality of services	11.8	1.5	0.0
No money	5.9	10.3	50.5
No insurance card	1.9	0.0	5.2
Distance	0.0	1.5	0.0
Illness not serious	23.5	30.9	11.3
No one to leave at home/farm	3.9	10.3	1.0
Had my own medicine/home remedies	43.1	38.2	26.8
Other	7.4	9.8	5.2

***ρ<0.01; based on Chi square test.

#### ***Choice of Provider***

**Figure 1:**
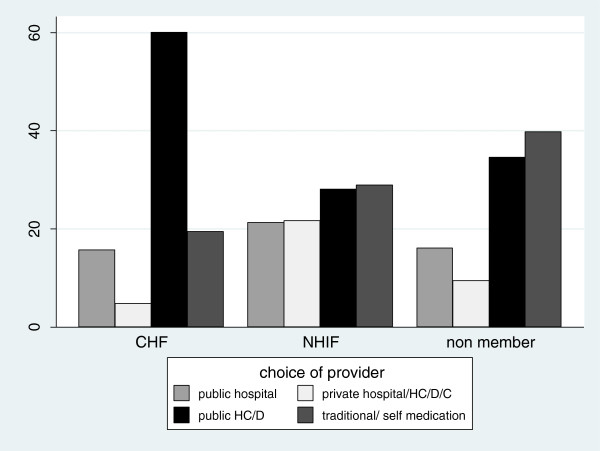
Choice of provider by membership status, conditioned on reporting illness (%).

The most common choice of provider was the public health centre/dispensary; 43% of all individuals seeking care chose this source, followed by traditional healer/self medication (28%), public hospital (18%) and private facilities (12%). About 22% of NHIF members who sought care for an illness went to a public hospital, compared to 16% of CHF members and 16% of non-members. A higher proportion of non-members opted for traditional healers/self medication (40%), compared to that of NHIF members (29%) and CHF members (20%, ρ<0.05). Figure 1 illustrates the different choice patterns by membership status. It shows that 60% of CHF members sought care from public health centre/dispensaries, compared to 28% of NHIF members and 35% non-members (ρ<0.05). Private facilities were preferred by 22% of NHIF members, 5% of CHF members and 10% of non-members.

#### ***Reasons for choice of provider***

Reasons for choice of provider are categorised into those for choice of formal care and those for choice of alternative forms of care (Table 4). Good quality (availability of drugs, laboratory tests, staff and most likely to find a doctor) was the main reason for seeking care from a district hospital, reported by 41% of non-members and NHIF members who used this provider. In contrast, 52% of CHF members who sought care from a public hospital said it was the only facility nearby (ρ<0.05). Quality reasons were also the most important for those who sought care from a private facility, reported by 73% of NHIF members, 50% of CHF members and 65% of non-members (ρ<0.05).

**Table 4:** Reasons for choice of care by membership status and provider, conditioned on seeking care (%)

**Variable**	**NHIF N = 171**	**CHF N = 237**	**Non-members N = 128**
District hospital (N = 130)***			
Good quality	40.8	15.2	41.3
Only facility available nearby	34.7	52.2	41.2
Insurance card accepted	20.1	30.4	NA
Exemption	NA	NA	8.8
Private Health centre/Dispensary (N = 85)***			
Good quality	72.6	50.0	65.0
Only facility available nearby	17.7	42.9	30.0
Insurance card accepted	5.9	0.0	NA
Exemption	NA	NA	0.0
Public Health center/Dispensary (N = 315)***			
Good quality	21.2	5.1	17.8
Only facility available nearby	60.6	64.2	75.3
Insurance card accepted	18.2	30.6	NA
Exemption	NA	NA	2.7
Choice of alternative care (traditional healer/self medication, N = 209)**			
	**N = 58**	**N = 51**	**N = 70**
No money	5.2	11.8	21.4
Distance	17.2	13.7	8.6
More likely to get treatment	43.1	47.1	27.1
Poor quality of services at formal facility	8.6	7.8	12.9
No insurance card	0.0	3.9	7.1
Illness not serious	8.6	3.9	2.7
Recognise symptoms, familiar with drugs	6.9	3.9	14.3
Other	8.6	7.8	5.7

**ρ<0.05; ***ρ<0.01; based on Chi square test.

Percentages may not add up to 100% since only the 3 main reasons are presented.

The main reason for seeking care from public primary care facilities is  only facility available nearby’, reported by 61% of NHIF members, 64% of CHF members and 75% of non-members who sought care from this source. Of particular interest is the importance of insurance for CHF members, reflected by about 30% whose choice of provider was based on the ability to use their insurance card, compared to 20% and 18% of NHIF members.

Among those who sought alternative forms of care, the main reason reported was that they were more likely to receive treatment from this source rather than from formal sources of care (NHIF 43%, CHF 47%, non-members 27%, ρ<0.05).

### Multivariate results

Table 5 presents results from the multinomial logit model specification.

**Table 5:** Multinomial logit estimation results, (public health centre/dispensary as reference)

**Variable**	**Public hospital**		**Private hospital**		**Traditional/self**	
	**Coeff.**	**Robust Std Error**	**Coeff.**	**Robust Std Error**	**Coeff.**	**Robust Std Error**
**Age (0–5**^**a**^**)**						
6-14	-0.241	0.369	0.113	0.437	0.340	0.302
15-49	0.116	0.340	0.693*	0.413	0.836**	0.304
50-69	0.707	0.548	0.131	0.737	1.010**	0.465
60+	-1.367	1.043	-0.363	1.103	0.648	0.579
**Sex (male**^**a**^**)**						
Female	0.023	0.260	0.250	0.299	-0.142	0.200
**Education level (no education**^**a**^**)**						
Up to Primary	0.437	0.540	0.378	0.638	0.480	0.405
Up to Secondary	-0.224	0.702	-0.964	0.908	-1.766**	0.646
Above Secondary	-1.346	0.567	-1.311	0.940	-2.191***	0.716
**Wealth status (lowest**^**a**^**)**						
Second	-0.533	0.594	-0.071	0.651	0.239	0.377
Third	0.360	0.490	0.214	0.693	0.811**	0.357
Fourth	1.008	0.626	1.295*	0.728	1.525**	0.553
Highest	1.675**	0.657	1.940**	0.746	1.313**	0.590
**Membership status (CHF**^**a**^**)**						
NHIF	0.037	0.679	1.664**	0.770	2.123***	0.574
Non-member	1.078**	0.411	1.594***	0.506	1.520***	0.307
**Residence (rural**^**a**^**)**						
Urban	3.721***	0.453	1.868***	0.446	1.046***	0.359
**Distance**	0.091	0.072	0.075	0.070	0.041	0.062
**Self reported morbidity (no illness**^**a**^**)**						
Acute	0.243	0.675	0.773	0.912	0.076	0.548
Chronic	1.695**	0.821	2.201**	1.022	0.980	0.677
**Perceived quality of care (poor**^**a**^**)**						
Availability of drugs	-0.791**	0.374	-1.085***	0.356	-0.358*	0.279
Adequacy of staff	0.196	0.371	0.388	0.326	0.386	0.300
**Constant**	-3.931***	0.894	-4.762***	1.380	-2.620***	0.733

*ρ<0.1; **ρ<0.05; ***ρ<0.01; ^**a**^base category.

#### ***The effect of enabling characteristics***

Persons from wealthier households are more likely to choose a public hospital, private facility and traditional healer/self medication over a public health centre/dispensary compared to those from relatively poorer households. The positive sign on the coefficient indicates that compared to CHF members, NHIF members are more likely to choose a private facility or traditional healer/self medication over a public health centre/dispensary. Likewise, non-members are more likely than CHF members to choose a public hospital, private health facility and a traditional healer/self medication over a public health centre/dispensary. Urban residents are more likely than rural residents to choose a public hospital, private facility and self medication over a public health centre/dispensary, regardless of health insurance status. Individuals are more likely to travel long distances to seek care from a public hospital rather than a public health centre/dispensary.

#### ***The effect of need characteristics***

Individuals reporting chronic illness are more likely to choose a public hospital or a private facility over a public health centre/dispensary. Results indicate that acute illness is not a significant determinant of choice of provider.

## Discussion

This paper examined the effects of fragmented risk pooling on health care seeking behaviour and utilisation of CHF and NHIF members and non-members. Results suggest that the insured are more likely to seek care and less likely to experience delays compared to non-members. Lack of money to pay for treatment is a significant barrier to seeking care for non-members but not for CHF and NHIF members, since this was the main reason for delays in seeking care or not seeking care at all reported by non-members. This suggests that generally health insurance does improve access to health care by reducing the financial barriers associated with utilisation of health services and is consistent with results reported by Jutting [37], Bronwyn et. al [40], Mensah et al. [41] and SHIELD [42]. However, members of the NHIF are more likely than CHF members to seek care for an illness and are also less likely to delay seeking care. This variation corroborates findings of Ekman [4] in Jordan who reported a higher probability of seeking care among members of the Ministry of health insurance programme compared to other programmes. The variation between CHF and NHIF members can be explained by the fact that compared to CHF members, NHIF members are more likely to live near a health facility and have a wider choice of providers compared to CHF members.

Differences were also found in relation to the choice of provider. Compared to CHF members, NHIF members are more likely to choose a private facility, traditional healer or self medication over a public health centre or dispensary. Although not significant, results also show that NHIF members are more likely than CHF members to choose a public hospital rather than a public health centre or dispensary. Good quality (availability of drugs, laboratory tests, staff and most likely to find a doctor) was the main reason for seeking care from a district hospital and private facility. District hospitals and private facilities are usually relatively better equipped and are more likely to have drugs than dispensaries or health centres [25]. Moreover, a non-functional referral system and no gate-keeping makes it easier for patients to bypass primary care facilities to seek care from hospitals [43,44]. Given that the NHIF coverage extends to all levels of care and to private accredited facilities, it is not surprising that members chose providers on the basis of perceived better quality of care. A study in Indonesia examining the effects of mandatory insurance on access to care also found a positive effect of the Askes (for public employees) on the use of public providers, while the Jamostek (for private employees) had a positive effect on use of both public and private providers. This was attributed to the differential benefit packages by the two funds [45].

The main reasons for choice of provider among CHF members were  only facility available nearby’ (64%) and  insurance card accepted’ (31%), while quality reasons were reported by only 5% of CHF members. This implies that CHF members will choose a provider that is nearby and where they can use their insurance card to pay for services regardless of quality of care offered. The CHF benefit package is limited to primary level care offered at health centres and dispensaries, and CHF registration occurs at the health facility, usually one that is nearest to where the member lives. Hence it is possible that members link their insurance entitlements to the facility where they registered making this facility the most logical choice. Therefore, unlike NHIF members, for CHF members, choice of provider involves a trade-off between claiming their entitlements from providers who may not necessarily provide the desired quality of care and forgoing their entitlements by paying out-of pocket for perceived superior quality. This may make health insurance an unattractive product, reducing the likelihood of re-enrolment of CHF members and negatively influence non-members’ decision to join the fund.

The variation in health seeking behaviour and choice of provider between the CHF and NHIF members is a reflection of the effects of a fragmented health insurance system. The lack of a standardised benefit package for the members of the two funds implies that while both CHF and NHIF members have better potential access, the quality and quantity entitled to respective members differs. In other words the whether, when, where health care is sought and the quality and quantity of health services received depends on health insurance affiliation. Given the lower revenue raising capacity of CHF compared to the NHIF, it also means that access to health care is based on ability to pay rather than on need for the services. This goes against the principle of equity of access and calls for mechanisms that will promote broader risk sharing and redistribution across the two schemes and a standardised benefit package. In this way for a given need, insured individuals will enjoy the same degree of access to health services regardless of insurance affiliation or ability to pay.

One of the goals of expanding health insurance in the Tanzanian health system was to eventually achieve universal coverage and universal access to health care. For a system to be truly universal it has to be equitable, granting access to the same range of services for all based on need while requiring payment for these services based on their income [46,47]. This can only be achieved in a health insurance system that is redistributive, such that there is risk sharing and cross-subsidisation across insurance schemes. Expanding health insurance to cover all population groups without redistributive mechanisms may achieve universal coverage but also create a system that does not support equity. Our results have shown the inequalities in access between the CHF and NHIF members.

The expansion of health insurance is a reflection of commitment by the Tanzanian government to achieve universal coverage. What remains is the development of policy framework and design issues that will promote redistribution and cross-subsidisation across the schemes in order to create a health insurance system that is universal and equitable. Creating a standardised benefit package is a crucial step in promoting equitable access to health services. The requirement for a standardised benefit package lies in the link between the package, risk structure and expected health expenditures of a scheme. Without a standardised benefit package, redistribution across schemes will reward those with more comprehensive packages at the expense of schemes with fewer benefits, perpetuating rather than reducing inequity.

In the Andersen behavioural model of health care utilisation, enabling characteristics such as income and health insurance are described as those that are necessary but not sufficient for utilisation [48]. Adequate health infrastructure capable of delivering quality health services has been mentioned as one of the pre-requisites of successful implementation of health insurance [49-51] and achievement of a truly universal health system [52]. The same arguments have been raised by Robyn et. al [3], who found that the effect of health insurance on health seeking behaviour in Burkina Faso was limited, owing to poor quality of services. This means that achieving basic coverage of the population is meaningless when this coverage does not guarantee access to services of an adequate quality. Our findings show that the extent to which health insurance promotes health care utilisation is dependent on the quality of services offered at formal care providers. When patient expectations are not met, it is likely that they will seek alternatives even though it means they have to forgo their insurance benefits. This has been elucidated by our results, which show that the majority of CHF and NHIF members who sought care from private facilities and from alternative sources (self-medication/traditional healer) did so due to perceived inferior quality in public facilities. Given the voluntary nature of CHF, and its potential for covering the majority of the population, quality improvements in health services are important to encourage enrolment. Scaling up enrolment into CHF is important for the redistributive potential of the scheme and ultimately of an integrated health insurance system.

## Conclusion

This paper examined the effects of fragmented risk pooling on health care seeking behaviour and utilisation of CHF and NHIF members and non-members. Specific areas that were examined were the decision to seek care when ill and the timing and choice of health service providers. The findings of this study provide lessons for policy makers in low- and middle-income countries where multiple health insurance funds have been established to achieve universal coverage. In particular, addressing the challenges of limited risk sharing and cross-subsidisation across multiple health insurance funds remains crucial for equitable access. These results confirm the importance of reducing fragmentation in risk pooling arrangements, creating the opportunity for risk and income cross-subsidisation that will also promote the development of a standard benefit package.

Perceived poor quality of health limits the degree to which the objectives of improved access and financial protection can be achieved. Furthermore, poor quality of health services serves as a deterrent for enrolment into voluntary funds, which often represent crucial elements for broadening redistribution and cross-subsidisation to promote equity.

## Endnotes

^a^Principal member is the contributing member of the NHIF, usually the head of household or spouse.

^b^In Tanzania, districts are organised into divisions, which in turn are divided into wards. Within each ward, there are a number of villages, which are also divided into hamlets. Depending on the ward and health infrastructure, one health facility may have a catchment area of one or more villages.

^c^Each village has a list of all households registered at the office of the Village Executive Officer. This list is broken down by hamlet but does not show membership status.

## Abbreviations

CHF: Community Health Fund; NHIF: National Health Insurance Fund; TIKA: Tiba kwa Kadi (urban equivalent of CHF); SHIB: Social Health Insurance Benefit; NSSF: National Social Security Fund; TDHS: Tanzania Demographic and Health Survey; PCA: Principal Components Analysis; MNL: Multinomial logit; IIA: Independence of Irrelevant Assumptions; RAS: Regional Administrative Secretary; DAS: District Administrative Secretary.

## Competing interests

The Authors declare no competing interests.

## Authors’ contributions

ENC was involved in study design, training of research assistants, review of data collection tools, data collection, data entry, data analysis and drafting the manuscript, revising and writing of the final manuscript. PGMM, UE, ADK and KH were involved in study design, review of data collection tools, data analysis and revising the manuscript. All the authors have read and approved the final version of the manuscript.
